# Prolonged Systemic Inflammation Alters Muscarinic Long-Term Potentiation (mLTP) in the Hippocampus

**DOI:** 10.1155/2021/8813734

**Published:** 2021-01-13

**Authors:** Efrat Shavit-Stein, Amir Dori, Marina Ben Shimon, Shany Guly Gofrit, Nicola Maggio

**Affiliations:** ^1^Department of Neurology, The Chaim Sheba Medical Center, Ramat Gan, 52621 Tel HaShomer, Israel; ^2^Sackler Faculty of Medicine, Tel Aviv University, 6997801 Tel Aviv, Israel; ^3^Sagol School of Neuroscience, Tel Aviv University, 6997801 Tel Aviv, Israel

## Abstract

The cholinergic system plays a fundamental role in learning and memory. Pharmacological activation of the muscarinic receptor M1R potentiates NMDA receptor activity and induces short-term potentiation at the synapses called muscarinic LTP, mLTP. Dysfunction of cholinergic transmission has been detected in the settings of cognitive impairment and dementia. Systemic inflammation as well as neuroinflammation has been shown to profoundly alter synaptic transmission and LTP. Indeed, intervention which is aimed at reducing neuroinflammatory changes in the brain has been associated with an improvement in cognitive functions. While cognitive impairment caused either by cholinergic dysfunction and/or by systemic inflammation suggests a possible connection between the two, so far whether systemic inflammation affects mLTP has not been extensively studied. In the present work, we explored whether an acute versus persistent systemic inflammation induced by LPS injections would differently affect the ability of hippocampal synapses to undergo mLTP. Interestingly, while a short exposure to LPS resulted in a transient deficit in mLTP expression, a longer exposure persistently impaired mLTP. We believe that these findings may be involved in cognitive dysfunctions following sepsis and possibly neuroinflammatory processes.

## 1. Introduction

The cholinergic system plays a fundamental role in brain functions. Cholinergic neurons are present in the cortex and in the hippocampus and regulate cognitive functions via nicotinic and muscarinic receptors [1]. While recent studies point towards its role in attention and cognitive control, the role of the cholinergic system in learning and memory is well established. Activation of the acetylcholine (Ach) muscarinic receptor potentiates NMDA glutamate receptors [2]. Pharmacological activation of the muscarinic receptor M1R potentiates NMDA receptor activity [3] and induces short-term potentiation at the synapses [4, 5]. The muscarinic signaling affects GABAergic interneurons as well, modulating long-term potentiation (LTP) and affecting hippocampal memory process [6]. Defects in the muscarinic signaling damage cognitive functions. Mutant mice lacking M1R have an altered spatial memory, suggesting a role of this receptor on memory process involving a cortex-hippocampus interaction [7]. Memory processes are affected by stress [8]. Repeated, unpredictable, stressful events have a long-term effect on the muscarinic potentiation (mLTP), in a steroidal hormones depended manner [9]. This data supports the involvement of the cholinergic system in stress-related memory dysfunction. Neuronal cholinergic system malfunctioning is evident in neuronal diseases with impaired memory such as Alzheimer's diseases (AD) [10]. AD, the most common dementia, affects 3% of the population aged 65-74 and almost 50% above the age of 85 [11]. AD is characterized by a constellation of symptoms, the hallmark of which is cognitive and behavioral impairment, which may involve impaired ability to process new information [12]. Cholinergic neurons' involvement led to the implantation of choline esterase inhibitors as part of the pharmacological treatment of AD. Indeed, the choline esterase inhibitor Donepezil improves cognitive function in AD patients [13]. Increasing evidence suggests that AD pathology also involves neuroinflammation processes [14].

Systemic inflammation, as manifested by elevation of systemically circulating inflammatory factors, is related to cognitive dysfunction in both delirium and dementia [15]. Furthermore, levels of IL-6, a reliable biomarker of systemic inflammation, are inversely related to hippocampal volume in healthy adults [16], suggesting that inflammation may affect memory outside of disease context. A prior neuronal cholinergic dysfunction is related to higher susceptibility for systemic inflammation in both humans [17] and animal modes [18].

Lipopolysaccharide (LPS) is a bacterial endotoxin known to induce systemic inflammation as well as brain neuroinflammation in animal models. Indeed, systemic injections of LPS lead to elevation of inflammatory factors in the brain [19–21]. Previous publications show that the injection of LPS leads to cognitive impairments [22, 23], and its injection in existing neurodegenerative disease may worsen cognitive function and accelerate disease progression [24].

Neuroinflammation is a pathological common pathway in a variety of diseases including such with impaired cognition [25–29], and its inhibition may cause an improvement in disease symptoms [30, 31], marking it as an important target for intervention.

Cognitive impairment caused by cholinergic dysfunction and by systemic inflammation suggests a possible connection between the two. So far, whether systemic inflammation affects mLTP has not been extensively studied. In the present work, we explore whether an acute versus persistent systemic inflammation induced by LPS injections would differently affect the ability of hippocampal synapses to undergo mLTP. Interestingly, while a short exposure to LPS resulted in a transient deficit in mLTP expression, a longer exposure persistently impaired mLTP. We suggest that these findings may be involved in cognitive dysfunctions following sepsis and possibly neuroinflammatory processes.

## 2. Methods

### 2.1. Mice and Treatments

Animal handling and experimental procedures were approved by the Institutional Animal Care and Use committee which adheres to the national laws and NIH rules (#694/11). As previously published [32], two-month-old Balb/c mice underwent two different treatment protocols. In one protocol, mice were exposed to intraperitoneal injections (*i.p.*) of LPS (1 mg/Kg) twice a week for a week (short treatment). In the other one, animals received LPS injections for a month (twice a week; long treatment). In order to assess whether the activation of the immune system prior to LPS administration would affect LTP, we exposed some of the animals to a single Complete Freund's Adjuvant (Adj) injection (diluted 1 : 1 in saline, 100 microliter total volume/injected mouse) 24 hours prior to the beginning of the LPS treatment. The rationale of exposing animals to LPS+Adj is due to the fact that Adj is known to enhance the inflammatory response and opens the blood-brain barrier [33]. In total, we had four groups of animals (*n* = 9 animals/group at each time point, one slice/animal for each pharmacological treatment) undergoing either a short or a long treatment. These included a group treated only with LPS (LPS) and a group treated only with adjuvant (Adj), and a third group was injected with Adj prior to LPS treatment (LPS+Adj) and untreated control animals.

### 2.2. Electrophysiology in Brain Slices

Extracellular recordings in acute slices prepared from dorsal hippocampus were performed as previously described [34, 35]. Following anesthesia with ketamine/xylazine (100 mg/kg and 10 mg/kg, respectively), animals were rapidly decapitated and 400 *μ*m hippocampal slices were prepared using a vibroslicer (NVSLM1 vibroslice, World Precision Instruments, Sarasota, FL, USA). Slices were incubated for 1.5 h in a humidified, carbogenated (5% CO_2_ and 95% O_2_) gas atmosphere at 33 ± 1°C and were perfused with ACSF [containing (in mM) 124 NaCl, 2 KCl, 26 NaHCO_3_, 1.24 KH_2_PO_4_, 2.5 CaCl_2_, 2 MgSO_4_, and 10 glucose, pH 7.4] in a standard interface chamber. Recordings were made with a glass pipette containing 0.75 M NaCl (4 MOhm) placed in the stratum radiatum of CA1 as described previously [34, 35]. Input-output curves were acquired from each slice prior to experimental assessment. Responses were digitized at 5 kHz and stored on a computer. Spike 2 software (Cambridge Electronic Design, Milton, Cambridge, England) was used for data acquisition. Data are reported as means ± SEM. Where appropriate, statistical analysis was performed with analysis of variance (ANOVA) followed by *post hoc* Tukey's comparisons.

## 3. Results

### 3.1. A Short Exposure to LPS Transiently Affects LTP Expression in the Hippocampus

**Figure 1:**
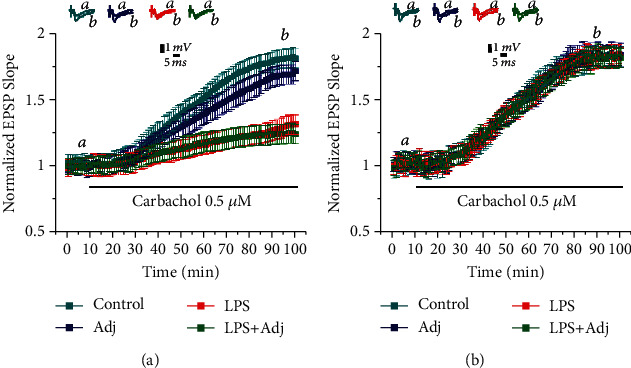
LPS transiently affects mLTP after a short treatment. (a) mLTP is impaired in animals treated with LPS and LPS+Adj for a week. (b) Upon interruption of the treatment for a week, mLTP is restored in LPS and LPS+Adj animals. Further details of the results and the statistical comparisons are described in “Results.” In each panel of data, the top traces are sample illustrations of original records before (a) and after (b) the application of 0.5 *μ*M carbachol.

It has been long known that application of 0.5 *μ*M of carbachol to ACSF bath induces a slow-onset LTP in hippocampal slices CA1 [36] called mLTP. In our hand, application of 0.5 *μ*M carbachol induced a potentiation that reached a plateau level of 1.80 ± 0.074 (at 90 min, *n* = 9 slices; Figure 1(a)). In order to investigate whether a short treatment with LPS would affect mLTP, we exposed animals to LPS injections at alternate days for a week (a total of 2 injections). Hippocampal slices from these animals were then processed for electrophysiology, and carbachol was applied to the ACSF bath (Figure 1(a)). Interestingly, hippocampal slices from these animals expressed a lower level of mLTP reaching values of 1.28 ± 0.075 (*n* = 9 slices). These values were similar to those reached by slices of animals treated with LPS+Adj (1.22 ± 0.052, *n* = 9 slices), while slices from animals treated with Adj alone did not differ from controls (1.78 ± 0.054, *n* = 9 slices; Figure 1(a)). Strikingly, LPS with or without Adj evoked only transient changes in mLTP. One week after termination of this short treatment, no changes in mLTP were detected in slices from animals treated with LPS or LPS+Adj compared to controls (1.78 ± 0.046 and 1.81 ± 0.037, *n* = 9, respectively; Figure 1(b)). A two-way ANOVA which is aimed at quantifying the effects of duration of LPS treatment (factor a) and the different groups of animals exposed to them (factor b) revealed an overall significant statistical difference for factor a (*F* (1, 64) = 45.11, *p* < 0.0001) and for factor b (*F* (3, 64) = 14.91, *p* < 0.001) as well as a significant interaction between the two (*F* (3, 64) = 19.46, *p* < 0.001). Overall, these results indicate that a short exposure to LPS or LPS+Adj both causes only a transient reduction in the ability to evoke mLTP in the hippocampus.

### 3.2. Prolonged Exposure to LPS+Adj Resulted in a Persistent Deficit in mLTP

**Figure 2:**
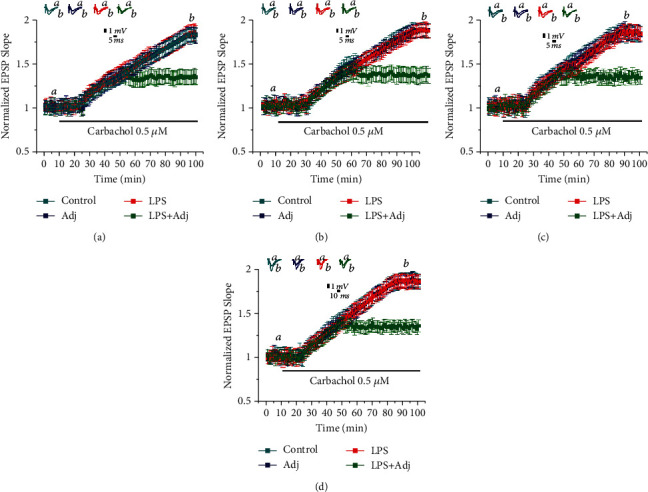
A prolonged treatment with Adj and LPS persistently impairs LTP in the hippocampus. Halting the treatment with Adj+LPS after a month resulted in a persistent impairment of mLTP checking at (a) 1 day, (b) 1 week, (c) 1 month, and (d) two months after treatment interruption. Further details of the results and the statistical comparisons are described in “Results.” In each panel of data, the top traces are sample illustrations of original records before (a) and after (b) the application of 0.5 *μ*M carbachol.

Systemic inflammation affects brain function [15]; however how prolonged systemic inflammation might affect the ability to evoke mLTP has not been addressed yet. Therefore, we exposed animals to a longer treatment protocol where animals received either LPS or LPS+Adj for one month at alternate days. Muscarinic LTP was then evaluated at one day, one week, one month, and two months after termination of treatments. Interestingly, prolonged LPS treatment did not result in a continuous reduction of mLTP (Figure 2). Slices from animals treated with LPS alone reached mLTP levels at 1 day (1.77 ± 0.044 at 90 min, *n* = 9 slices; Figure 2(a)), 1 week (1.79 ± 0.044, *n* = 9 slices; Figure 2(b)), 1 month (1.76 ± 0.063, *n* = 9 slices; Figure 2(c)), and 2 months (1.78 ± 0.038, *n* = 9 slices; Figure 2(d)) after treatment which were similar to control. However, a different phenomenon occurred when adjuvant was administered prior to prolonged LPS treatment. In the LPS+Adj group, remarkably, mLTP was not restored to normal levels even 2 months after termination of treatment. Slices from the LPS+Adj group failed to express mLTP at each of the evaluated time point (respectively 1.31 ± 0.063 at 1 day, 1.29 ± 0.048 at 1 week, 1.23 ± 0.043 at 1 month, and 1.29 ± 0.043 at 2 months, *n* = 9 slices; Figure 2). To quantify the effects of duration of LPS treatment (factor a) and the different groups of animals exposed to them (factor b), we ran a two-way ANOVA which overall did not reveal a significant statistical difference for factor a (*F* (3, 128) = 0.4965, *p* = 0.68); however, a statistical difference was found for factor b (*F* (3, 128) = 109.2, *p* < 0.0001). Strikingly, a post hoc Tukey analysis revealed a significant difference between the LPS+Adj group to all other animal groups at the tested time points (values taken at 90 min, *p* < 0.001). In summary, these experiments show that prolonged treatment with LPS in animals previously exposed to Adj induces a persistent reduction in mLTP that could not be reestablished to normal levels 2 months after termination of LPS treatment.

## 4. Discussion

In this study, we investigated the effect of systemic inflammation through injections of i.p. LPS on the ability to evoke mLTP in hippocampal slices. Our finding shows that systemic inflammation leads to different outcomes on mLTP depending on its duration. A short-lasting inflammation resulted in transient reduction of mLTP. In contrast, mLTP was disrupted for a longer period in slices from animals following prolonged (one month) exposure to LPS, long after this exposure was terminated, if they were previously treated with Adj. In contrast, mLTP was normal in animals with prolonged exposure only to LPS.

The results obtained in this study are similar to those presented in a previous study that was performed in our laboratory [32]. In the latter, we evaluated the role of systemic inflammation in evoking tetanic-induced LTP in animals treated with LPS with or without Adj exposure. Similarly to the present findings, we found that tetanic-induced LTP is transiently impaired following a short treatment with LPS, although a persistent impairment in tetanus-induced LTP was found when LPS treatment was prolonged for a month in animals previously exposed to Adj. The fact that both studies reached similar conclusions points to the possibility that systemic inflammation may trigger in the brain-related mechanism possibly acting at the postsynaptic side. In this respect, both tetanus-induced LTP and mLTP share a common postsynaptic downstream mechanism based on NMDA receptor potentiation. Therefore, it is possible to speculate that systemic inflammation may likely depress NMDA-induced currents thus resulting in impaired forms of both LTP. Although both studies have not addressed the issue of how systemic inflammation may affect NMDA receptor-mediated currents, it is feasible to hypothesize a pivotal role of neuroinflammation in this phenomenon. It has been known that systemic inflammation may lead to neuroinflammation [37, 38]. In this setting, the excessive release of inflammatory cytokines, such as IL-1, IL-6, and TNF alpha in the CNS, may directly affect NMDA function and thus depress LTP. An additional mechanism that might explain the observed phenomena may also relate to the specific effects of neuroinflammation on cholinergic activity. It has been indeed shown that M1R mRNA is downregulated upon a LPS challenge [39], thus implicating a possible lower mLTP in this setting.

Further experiments should be performed to evaluate such hypotheses. A puzzling finding of our study lies on the evidence that a prolonged LPS treatment alone does not alter LTP; however, exposing animals to a combination of LPS+Adj resulted in a lower level of mLTP which remained for longer time scale even after the treatment was terminated. We have not addressed the possible mechanism of this finding; however, we speculate that two different mechanisms may underlie the described phenomena. A prolonged LPS treatment alone may result in a tolerance of the brain such that a repeated exposure to a given concentration of the same molecule fails to trigger an adequate response [40]. Alternatively, a tolerance of the blood-brain barrier to LPS may result in lower concentration of the molecule reaching the brain and thus in a reduced response over time. In contrast, adjuvant pretreatment may “prime” the immune system as well as promote a long-lasting BBB breakdown [33], resulting with LPS triggering an effective long-lasting neuroinflammation that might persistently modify brain function and thus result in a lasting modification of mLTP. Finally, it is also interesting to speculate the possibility that at longer time scales, Adj may add a synergistic effect on LPS and thus result in a possible BBB disruption which may underlie the long-term effects on mLTP of the Adj+LPS treatment.

Sepsis has long been known to alter cognition in acute clinical settings and cause delirium [41]. We and others have shown that LTP impairments following sepsis may underlie the physiological mechanism of this phenomenon [32]. Drugs enhancing cholinergic and M1 activity have been hypothesized to be beneficial for treatment of septic delirium [42, 43]. In this context, our study further supports the conclusion that cholinergic LTP is affected in the context of systemic inflammation and thus strengthen the conclusion that cholinergic modulation may be beneficial in order to prevent sepsis-induced delirium.

mLTP impairment has also been hypothesized to be one of the first pathophysiological steps towards developing cognitive decline in the context of Alzheimer's dementia [44–46]. Recent evidences have pointed to a fundamental role of neuroinflammation in the pathogenesis of this disease [14]. In this context, neuroinflammation may affect mLTP in the early stages of the disease. Therefore, it might be interesting to evaluate whether contrasting a reduction of mLTP may correlate with a delay in cognitive decline at the early stages of the disease.

In conclusion, our study shows that systemic inflammation affects muscarinic LTP and leads to different outcomes depending on treatment duration. Although, conclusions suffer of limitations due to the fact that we cannot clearly indicate the mechanisms underlying the observed phenomena, we believe that this research advances the current understanding of how long-lasting systemic inflammation affects mLTP. This may propose possible strategies in order to prevent cognitive impairment in predisposed individuals.

## Data Availability

All data will be available upon reasonable request.
